# The Usefulness of 3-Dimensional Virtual Simulation Using Haptics in Training Orotracheal Intubation

**DOI:** 10.1155/2013/534097

**Published:** 2013-09-19

**Authors:** Dong Hoon Lee, Jae Gyu Kim, Chan Woong Kim, Chang Ha Lee, Jae Hee Lim

**Affiliations:** ^1^Department of Emergency Medicine, College of Medicine, Chung-Ang University, Chung-Ang University Hospital, 224-1 Heoukseok-dong, Dongjak-gu, Seoul 156-757, Republic of Korea; ^2^Department of Internal Medicine, College of Medicine, Chung-Ang University, Chung-Ang University Hospital, 224-1 Heoukseok-dong, Dongjak-gu, Seoul 156-757, Republic of Korea; ^3^Chung-Ang Institute for Simulation, Education, and Research, College of Medicine, Chung-Ang University, Chung-Ang University Hospital, 224-1 Heoukseok-dong, Dongjak-gu, Seoul 156-757, Republic of Korea; ^4^Medical Education of Clinical Medicine, College of Medicine, Chung-Ang University, Chung-Ang University Hospital, 224-1 Heoukseok-dong, Dongjak-gu, Seoul 156-757, Republic of Korea; ^5^School of Computer Science and Engineering, Chung-Ang University, 224 Heoukseok-dong, Dongjak-gu, Seoul 156-756, Republic of Korea; ^6^Graduate School, College of Medicine, Chung-Ang University, 224 Heoukseok-dong, Dongjak-gu, Seoul 156-756, Republic of Korea

## Abstract

*Objectives*. Airway control is the most critical treatment. The most common and basic method of endotracheal intubation is orotracheal intubation. To perform accurate and rapid tracheal intubation, appropriate education and training are required. We developed the virtual simulation program utilizing the 3-dimensional display and haptic device to exercise orotracheal intubation, and the educational effect of this program was compared with that of the mannequin method. *Method*. The control group used airway mannequin and virtual intubation group was trained with new program. We videotaped both groups during objective structured clinical examination (OSCE) with airway mannequin. The video was reviewed and scored, and the rate of success and time were calculated. *Result*. The success rate was 78.6% in virtual intubation group and 93.3% in control group (*P* = 0.273). There was no difference in overall score of OSCE (21.14 ± 4.28 in virtual intubation group and 23.33 ± 4.45 in control group, *P* = 0.188), the time spent in successful intubation (*P* = 0.432), and the number of trials (*P* > 0.101). *Conclusion*. The virtual simulation with haptics had a similar effect compared with mannequin, but it could be more cost effective and convenient than mannequin training in time and space.

## 1. Introduction

Airway control is the first and most critical treatment. The “A” in the ABCs demands that no other action may take place until the airway is secure. If the airway is not secure, nothing can help the patient. The endotracheal intubation can be accomplished by a variety of methods. The method of choice will be dictated by physician preference and experience, the patient's condition, and the type of equipment available. Because all doctors with acute care responsibilities are expected to be competent in airway management, appropriate education and training were required [1]. In education of medical student, the teaching program was contained to train them to perform orotracheal intubation with mannequin. And the orotracheal intubation was a test among objective simulation constructive examination (OSCE) for Korean Medical License Examination.

Orotracheal intubation is basic technique in securing airway of patients [2]. But, actually, it was difficult for many medical doctors to conduct the procedure when emergent situation, because they had not been trained enough. Although other airway management techniques, such as supraglotic airway, were relatively easy and did not need large amount of effort and time to train the procedure these skills need specific device and these devices could not be always prepared. Therefore, there is a need to provide training in orotracheal intubation as the basic method of airway management. Generally, orotracheal intubation was educated using airway mannequin. However, it is hard for many medical personnels to be trained with mannequin because of limited time and space. We developed the 3-dimensional simulation program for medical students to exercise orotracheal intubation easily. And the educational effect of this computer program was compared with the traditional training method using airway mannequin. 

## 2. Method

This was a prospective randomized observational study and it was approved by the Chung-Ang University Hospital international review board. 

### 2.1. 3D Virtual Simulation Program

**Figure 1:**
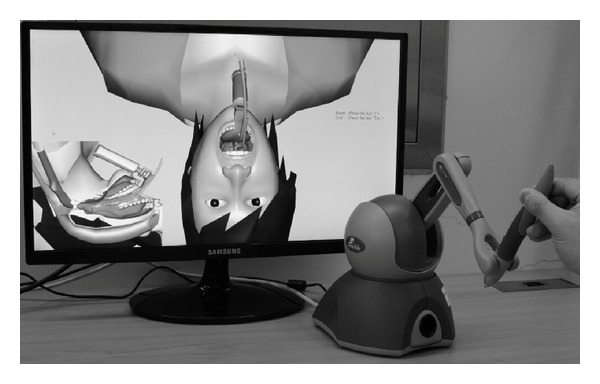
The virtual intubation program with 3-dimenstional display and haptic device.

We developed the computer program in which students could be trained to exercise orotracheal intubation in 3-dimentional simulation. This included the anatomy from nasopharynx to trachea. The anatomic reconstruction of program was done from the data of healthy volunteer who was male and 25 year old. Neck CT was examined by 256 channels MDCT and the high-resolution images were collected. These images were used to reconstruct to 3D image and the image of laryngoscope was emerged to produce the sequence that vocal cord was visualized by laryngoscope. Haptics was used to train in the handling technique of endotracheal tube (Figure 1). Due to using haptics, the students could not advance the endotracheal tube when they handled the tube to the wrong direction. When the tube was placed in trachea correctly, the program shows the success message in monitor.

### 2.2. Evaluation of the New Simulation Program

After informed consent, 29 undergraduate medical students were enrolled. They were 5th grade in 60-year medical school. Exclusion criteria were any previous experience with orotracheal intubation training. Students were randomized into two groups. Both groups were instructed about the anatomy of airway and method of orotracheal intubation from preparation to confirm the airway for 1 hour. And the control group exercised the procedure with airway mannequin (Laerdal Medical, Stavanger, Norway). Virtual intubation group was trained with haptic device and computer with 3D monitor in other place. Each group had no idea about the training method of the other group and they exercised the procedure for 1 hour. After 2 hours of resting time, we tested students by OSCE with airway mannequin. We videotaped both groups during OSCE, then reviewed and scored them, and the rate of success and time were calculated. We determined the success of intubation with tracheal placement of tube. The score from OSCE and data from video analysis were compared using Student's *t*-tests and *x*2-tests with SPSS 19.0 (SPSS, Chicago, IL, USA). A *P* value <0.05 was considered statistically significant. 

## 3. Result

There was no statistical difference in age and gender between the two groups. The success rate was 78.6% in virtual intubation group and 93.3% in control group (Table 1, *P* = 0.273). There was no difference between the two groups in overall score of OSCE (21.14 ± 4.28 in virtual intubation group and 23.33 ± 4.45 in control group, *P* = 0.188), the time spent to successful intubation (*P* = 0.423), and the number of trials (*P* = 0.101).

## 4. Discussion

Orotracheal intubation is the primary and preferred method of airway management. Every physician must master this skill. It was done mainly in emergency situations except anesthesia. In this situation, most physicians might be confused and hesitate to perform orotracheal intubation because they had not been trained enough and had a fear of failure and complications of the procedure. And endotracheal intubation itself can evoke a transient but marked response on manifested as hemodynamic changes [3]. Therefore, many medical schools and medical facilities had a curriculum to train students and houseman in airway management.

In medical schools students attend didactic lecture in orotracheal intubation and practice with airway mannequin. In order for orotrahceal intubation to success, it is important to visualize vocal cord and insert endotracheal tube inside the vocal cord to place the tube in tracheal. And it needs a lot of practice for accurate and rapid procedure. Mannequins have been used generally as a tool to teach invasive procedures to medical personnel and students. With airway mannequin, students exercise mainly the handling of laryngoscope to visualize vocal cord and advancing endotracheal tube to trachea. In addition to the experience of the actual handling of the device, they could practice the whole sequence of procedure from preparing patients and devices to confirming the security of airway. The simulation with mannequin is free to ethical problems. But it needs the mannequin and many other devices such as oral airway, bag valve mask, laryngoscope, and endotracheal tube. This equipment is expensive and could be destroyed during simulation. Many students could not be trained at the same time because the number of equipment was limited, so they could not have the chance to be trained the procedure enough.

We tried new educational device for orotracheal intubation utilizing a 3D monitor and haptic device. Using this program, students can understand the anatomy of the airway and learn to place the tube in the trachea. The previous educational method to train, orotracheal intubation used airway mannequins and fresh frozen cadaver. Using the cadaver provided real training, but it was not available for a large number of medical personnel [4]. The overall score of OSCE that evaluated the full process of intubation did not differ. Although the virtual intubation group did not actually handle the laryngoscope and endotracheal tube, they understood the anatomy of airway and succeeded repeatedly, resulting in no difference in the success rate. Previous studies reported that the 3D virtual simulation was effective in training specific procedure such as gastro-intestinal endoscopy and neurosurgery [5–10]. However, there was no study for virtual simulation to train orotracheal intubation. 

Virtual simulations in this study had several advantages. They are free of ethical problems because students do not practice with real patients. And it is cost-effective because computer with 3D monitor and haptic device can be used for a long time. It can be used for self-training on any computer without any additional equipment [11, 12]. On the other hand, airway mannequin may break down due to long-term use and is needed to change expensive consumable. For macrosimulation using mannequin, the large space such as simulation center is needed, but this program only requires smaller space with installed the computer and haptic device. For an educational aspect, students can each select the difficult step and practice only their part, making destruction learning possible.

## 5. Limitation

The 3D computer simulation used in this study was proto-type, so it was possible to train only handling the endotracheal tube in orotracheal intubation. Namely, one hand grasping the tube could be exercised. It could not train the other hand that grasps the laryngoscope to visualize vocal cord. This step is critical for successful orotracheal intubation. In this study, the students' failure in test was due to this step. In process of orotracheal intubation, the confirmation of correct placement of tube is important, and also the preparation of procedure preoxygenation of patient were important. However, this program could not train these steps. It is necessary to be supplemented because is inadequate.

## 6. Conclusion

The training of orotracheal intubation was necessary for medical people for airway management. The virtual simulation with 3-demsional display and haptic device had similar effect on training in the procedure compared with airway mannequin. And this program could be more cost effective and convenient for students to practice the procedure in time and space. Therefore, thee virtual intubation programs with 3-dimensional display and haptic device could be alternative to traditional training method with airway mannequin.

## Figures and Tables

**Table 1:** The comparison of the educational effect between two groups.

	Virtual 3D simulation	Mannequin simulation	*P* value
Score of OSCE	21.14 ± 4.28	23.33 ± 4.45	0.188
Result of simulation (%)			0.278
Success	11 (78.6)	14 (93.3)	
Failure	3 (21.4)	1 (6.7%)	
Time to success (sec)	234.29 ± 52.51	219.33 ± 46.54	0.423
Number of trial	1.79 ± 0.98	1.27 ± 0.59	0.101
